# Real-Time Temperature Correction of Medical Range Fiber Bragg Gratings Dosimeters

**DOI:** 10.3390/s23020886

**Published:** 2023-01-12

**Authors:** Marie-Anne Lebel-Cormier, Tommy Boilard, Luc Beaulieu, Martin Bernier

**Affiliations:** 1 Département de Physique, de Génie Physique et d’Optique, Université Laval, Québec, QC G1V 0A6, Canada; 2 Centre de Recherche sur le Cancer, Université Laval, Québec, QC G1R 3S3, Canada; 3 CHU de Québec Université—Laval et CRCHU de Québec, Québec, QC G1R 2J6, Canada; 4 Centre d’Optique, Photonique et Laser (COPL), Université Laval, Québec, QC G1V 0A6, Canada

**Keywords:** fiber dosimetry, FBG temperature correction, radiotherapy multi-points dosimetry

## Abstract

The interest in fiber Bragg gratings dosimeters for radiotherapy dosimetry lies in their (i) submillimeter size, (ii) multi-points dose measurements, and (iii) customizable spatial resolution. However, since the radiation measurement relies on the thermal expansion of the surrounding polymer coating, such sensors are strongly temperature dependent, which needs to be accounted for; otherwise, the errors on measurements can be higher than the measurements themselves. In this paper, we test and compare four techniques for temperature compensation: two types of dual grating techniques using different coatings, a pre-irradiation and post-irradiation temperature drift technique, which is used for calorimetry, and finally, we developed a real-time interpolated temperature gradient for the multi-points dosimetry technique. We show that, over these four tested techniques, the last one outperforms the others and allows for real-time temperature correction when an array of 13 fiber Bragg gratings spatially extending over the irradiation zone is used. For a 20 Gy irradiation, this technique reduces the measurement errors from 200% to about 10%, making it suitable for a radiotherapy dose range. Temperature correction for medical low-dose range dosimetry is a first in our field and is essential for clinical FBG dosimetry applications.

## 1. Introduction

Fiber Bragg gratings (FBGs) are well-established sensors that are used in various industries to measure temperature, stress, pressure, etc. [1,2,3,4]. Since FBG sensors respond to different physical perturbations (temperature, stress, pressure, etc.), the differentiation of these variables is a common challenge because they often vary simultaneously [5]. Hence, many techniques have been developed over the years to compensate for one variable or to measure two variables at once. The dual grating technique, which consists of using two FBGs having a different response to the varying physical perturbations, is commonly used to differentiate strain and temperature. To ensure a different response of both FBGs, different fiber cladding sizes [6], reflected wavelengths [7], grating types [8], fibers material [9], and inscription techniques (normal and reverse index FBGs) [10] can be used. A single grating can also be used to discriminate the signal of interest. For example, Echevarria et al. [11] measured the change in the Bragg wavelength associated with the first and the second diffraction order of an FBG to discriminate strain and temperature. Single and dual grating techniques have also been developed to discriminate temperature from pressure, curvature, load, or other parameters [12]. Since temperature is often the undesirable signal, another solution for this challenge is to develop a sensor that is insensitive to temperature [12]. For example, Osuch [13] demonstrated that, by applying a force to a chirped FBG written in a tapered optical fiber, it produces variable strain along it due to the variable optical fiber diameter along the grating, which leads to a change in its spectral width. In this case, since the temperature impacts only the reflected Bragg wavelength, measuring the response in width allows strain-independent measurements [14].

FBGs are also known to be affected by the presence of radiation, which makes the use of this type of sensor even more challenging for applications in the nuclear industry or in space [15]. For a high dose range, in the nuclear field, precisely controlling the temperature around the sensor has been shown to be an effective way to compensate ambient temperature variations [16]. It was achieved by placing the dosimeter in a thick-wall aluminum container having heating elements that are controlled by a proportional-integral-derivative controller. Thermocouples have also been used to monitor temperature variations during high dose range irradiation, which is then translated into reflected wavelength shift and can be subtracted from the measured wavelength shift to obtain solely the radiation associated signal [17,18]. Esposito et al. [19] have used thermocouples to compensate for ambient temperature variations, but they also placed the sensor in an insulated box to limit the variations.

We previously showed that Bragg wavelength shifts (BWS) of polymer-embedded FBGs are usable for medical range dosimetry [20]. They would be an excellent candidate especially for small field radiotherapy multi-points dosimetry [21] because of their small detection volume (around 0.01 ${\mathrm{mm}}^{3}$) compared to a commonly used ion chamber [22]. Olusoji et al. [23] have recently shown that FBGs written in a perfluorinated polymer could also be used for medical range dosimetry. Like other types of FBG sensors, FBG-based dosimeters are ambient temperature dependent, which means that temperature variations need to be accounted for; otherwise, the errors on measurements can be higher than the measurements itself. Unlike the nuclear field, to access radiotherapy treatment quality, the radiation fluence should stay undisturbed by the presence of the detector [24]. Hence, it is impossible to place metal in the treatment field to compensate for temperature variations as is generally carried out for the high dose range. Additionally, since the head of the linear accelerator is rotating around the detector to deliver the treatment, the detector should preferably be angularly independent of the radiation source location, which could prove hard to achieve for the dual grating technique for instance, since the reference grating should be placed in a different location than the measurement grating.

In this article, we set out to test four different techniques to compensate for the temperature variation occurring during medical low-dose range irradiation of polymer-embedded FBGs, which to our knowledge, has never been tried before. We tested two types of dual grating techniques using different coatings (uncoated FBG, polymethyl methacrylate (PMMA) coated FBG and polypropylene (PP) coated FBG). We also tested the pre-irradiation and post-irradiation temperature drift (PPD) technique with PMMA coated FBGs, which is commonly used for calorimetry [25]. Finally, we created a real-time interpolated temperature gradient for multi-points dosimetry (ITG) technique with PMMA coated FBGs. This last technique would be preferable for radiotherapy dosimetry since it allows real-time correction. We show that this last technique outperforms the others and allows for real-time temperature correction when an array of 13 FBGs spatially extending over the irradiation zone is used. For a 20 Gy irradiation, this technique reduces the measurement errors from 200% to about 10%, making it suitable for the radiotherapy dose range.

## 2. Materials and Methods

### 2.1. Theory

For a polymer-embedded FBGs dosimeter, we previously showed good accordance for BWS ($\Delta \lambda_{B}$) due to low-dose radiation response (*D*) between experimental data and theoretical model [21]:

(1) $$\frac{\Delta \lambda_{B}}{\lambda_{B}}=\left[\left(1-p_{e}\right)\left(\alpha_{c}-\alpha_{f}\right)\frac{A_{c}E_{c}}{A_{c}E_{c}+A_{f}E_{f}}\right]\frac{D}{c_{c}}+[\alpha_{f}+\xi ]\frac{D}{c_{f}}$$

in which $\lambda_{B}$ is the Bragg peak wavelength, $p_{e}$ is the photo-elastic coefficient, $\alpha_{c}$ and $\alpha_{f}$ are the coefficient of thermal expansion for the coating and for the fiber, $A_{c}$ and $A_{f}$ are the coating and the fiber cross-sectional areas and $E_{c}$, $E_{f}$ are Young’s modulus of the coating and of the fiber, $c_{c}$ and $c_{f}$ are respectively the coating and fiber mass heat capacity, and ξ is the thermo-optic coefficient. If we consider ambient temperature variation (ΔT) and set $\beta =\left(1-p_{e}\right)\left(\alpha_{c}-\alpha_{f}\right)\frac{A_{c}E_{c}}{A_{c}E_{c}+A_{f}E_{f}}$ for convenience, the total BWS can be expressed as follows:

(2) $$\frac{\Delta \lambda_{B}}{\lambda_{B}}=\left[\frac{\beta }{c_{c}}+\frac{\alpha_{f}+\xi }{c_{f}}\right]D+[\beta +\alpha_{f}+\xi ]\Delta T,$$

### 2.2. Dual Grating Technique

The dual grating technique consists of using two FBGs to create a system of two linear equations having two unknown parameters (*P* and $P^{\prime }$), which can be represented as follows:

(3) $$\left[\begin{array}{c} \Delta \lambda_{B1}/\lambda_{B1}\\ \Delta \lambda_{B2}/\lambda_{B2} \end{array}\right]=\left[\begin{array}{cc} K_{P1} & K_{P^{\prime }1}\\ K_{P2} & K_{P^{\prime }2} \end{array}\right]\left[\begin{array}{c} P\\ P^{\prime } \end{array}\right]$$

where KP1 and KP1′ are respectively the first and second parameter coefficients of the first fiber and KP2 and KP2′ are respectively the first and second parameter coefficients of the second fiber. This type of correction is effective only if the coefficient matrix, containing the four coefficients, is non-singular, which implies that $\frac{K_{P1}}{K_{P2}}\neq \frac{K_{P^{\prime }1}}{K_{P^{\prime }2}}$. For polymer-embedded FBG dosimeters, this equation becomes:

(4) $$\left[\begin{array}{c} \Delta \lambda_{B1}/\lambda_{B1}\\ \Delta \lambda_{B2}/\lambda_{B2} \end{array}\right]=\left[\begin{array}{cc} K_{D1} & K_{\Delta T1}\\ K_{D2} & K_{\Delta T2} \end{array}\right]\left[\begin{array}{c} D\\ \Delta T \end{array}\right]$$

where

(5) $$K_{D}=\left[\frac{\beta }{c_{c}}+\frac{\alpha_{f}+\xi }{c_{f}}\right]\;\mathrm{and}\;K_{\Delta T}=\left[\beta +\alpha_{f}+\xi \right]$$

Hence, to achieve the dual grating correction technique with a polymer-embedded FBG dosimeter, many approaches could be taken such as different polymer coating sizes, different types of polymer compositions, etc.

The precision at which both parameters can be determined will depend on the precision of the measured parameters and calibration [26]. The limitation generally rises when the coefficient matrix determinant is close to zero, which leads to significant errors and low accuracy [12].

### 2.3. Pre-Irradiation and Post-Irradiation Temperature Drift (PPD)

**Figure 1:**
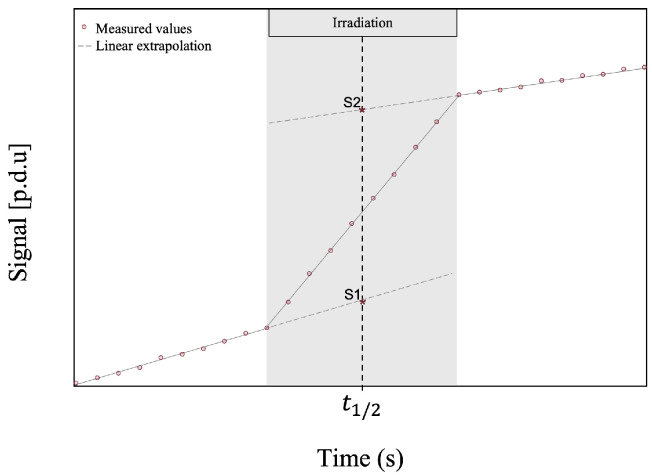
Example of pre-irradiation and post-irradiation temperature drift correction technique, for which two linear extrapolations are created, one with the pre-irradiation signal and one with the post-irradiation signal and evaluated (corresponding to S1 and S2 on the figure) at mid-irradiation time ($t_{1/2}$).

PPD technique is commonly used in calorimetry [27], which consists of measuring the rise in temperature generated by radiation interactions within a medium. Even if the temperature of the medium is kept relatively stable for calorimetry, the temperature variation produced by radiation is so small (around 0.0005 ${}^{\circ }C$ in water for a standard treatment dose of 2 Gy) that drifts in temperature are often measured in the medium itself. The traditional method to correct this type of temperature drift is the PPD technique. As shown in Figure 1, to obtain the signal of interest with this correction technique, two linear extrapolations are created, one with the pre-irradiation signal and one with the post-irradiation signal. These extrapolations are evaluated (corresponding to $S_{1}$ and $S_{2}$ in the figure) at mid-irradiation time ($t_{1/2}$) and subtracted ($S_{2,t_{1/2}}-S_{1,t_{1/2}}$) [25].

### 2.4. Real-Time Interpolated Temperature Gradient for Multi-Points Dosimetry (ITG)

The real-time interpolated temperature gradient technique consists of using parts of the multi-points FBG dosimeter as thermometers to correct ambient temperature variations during irradiation. The FBGs located outside the irradiation field measure minimal radiation doses, which is assumed to be zero for this temperature correction technique. For this technique, two main constraints were considered for optimal usage in radiotherapy: it should not add noise to the signal and it has to be usable in real-time. Considering this first constraint, we could not simply subtract point by point the reference signal to the dosimeters signal, which would add both signal noises. Hence, we decided to apply a rolling slope at every time point. In order to have this technique usable in real-time, only the signal preceding the time point of interest can be used. Since we glue the coating to the fiber with UV glue, the bonding strength may vary slightly for every FBG, which needs to be accounted for. This implies that a calibration is required. Therefore, the proposed ITG technique has three requirements: a pre-irradiation acquisition to execute the rolling slope, a calibration to account for the varying bonding strength and that part of the multi-points FBG dosimeter at both ends remains outside the irradiation field.

### 2.5. Experimental Setup

Unless stated otherwise, the polymer-embedded FBGs are made by machining a 200 μm wide groove halfway through a plate of the selected polymer and then, using a UV adhesive, gluing the fiber containing the FBGs inside of it. In this experiment, the FBGs are written directly through the polyimide-coating of the fiber (BF06160-02, OFS) using our femtosecond scanning phase mask writing setup, the details of which can be found in [28,29]. The use of a custom e-beam phase-mask with multiple uniform periods along its length, twenty spanning the 1500–1600 nm wavelength range in this experiment, facilitates the inscription of arrays of FBGs with precise spectral and spatial specifications.

The polymer-embedded FBGs are placed at a depth of maximum dose of 1.5 cm in a diffusing material (solid water) and are irradiated by a 10 × 10 cm${}^{2}$ 6 MV photon beam at a rate of 6 Gy/min, up to a dose of 20 Gy (200 s), on a radiation therapy accelerator (Clinac iX, Varian) [20]). Their wavelengths are first recorded at 1 kHz using a commercially available interrogator (si155, Micron Optics (now Luna Innovations)), and then, based on the assumption that the temperature variation of each FBG is negligible over 1 s, the recorded wavelengths are averaged each second to reduce the error of 1 pm provided by the manufacturer on every data point to 0.03 pm, a reduction by a factor of roughly 30 ($\sqrt{1000}$).

### 2.6. Dual Grating Technique

For the first dual grating technique, the detector is made of one 3 × 3 × 20 ${\mathrm{mm}}^{3}$ polymer-embedded (polymethyl methacrylate) fiber and a bare silica fiber. The bare silica fiber is not sensitive enough to detect radiation-induced temperature variation, hence it serves as an ambient temperature detector here ($K_{D2}=0$). For this technique, the calculated theoretical temperature coefficient ratio ($K_{T1}/K_{T2}$) is 6.74.

For the second dual grating technique, the detector used is made of two 3 × 3 × 20 ${\mathrm{mm}}^{3}$ polymer-embedded (PMMA and PP) FBGs. Again, the temperature coefficients are calculated theoretically, but the dose coefficients are accessed experimentally by averaging the response of two irradiations (while the ambient temperature was found constant) for both polymer-embedded FBGs. For this technique, the temperature coefficient ratio ($K_{T1}/K_{T2}$) is 1.52, and the dose coefficient ratio ($K_{D1}/K_{D2}$) is 1.18, which are different from one another as required. All theoretical coefficients are calculated with the constants presented in [21].

### 2.7. Pre-Irradiation and Post-Irradiation Temperature Drift (PPD)

For the PPD technique, the detector is made of a fiber having twenty 4 mm-long FBGs, equally distributed over 20 cm, which is embedded in a PMMA sheet having a 5.5 × 107.5 × 205 mm${}^{3}$ size. The classical correction method, presented previously (Figure 1), would provide only the corrected total irradiation signal, but to obtain the corrected signal in time we applied a slightly different method which is mathematically equivalent. We acquired 60 s before and after the irradiation and applied a linear regression to both pre-irradiation and post-irradiation signals. We calculated the average of both linear regressions’ slopes to obtain a corrected slope. This corrected slope is then subtracted from the irradiation signal for every time point. Once corrected, the 60 s pre-irradiation measurements are average and set to zero to allow fair comparison between each FBG. This technique was used in our previous work [20,21].

### 2.8. Real-Time Interpolated Temperature Gradient for Multi-Points Dosimetry (ITG)

**Figure 2:**
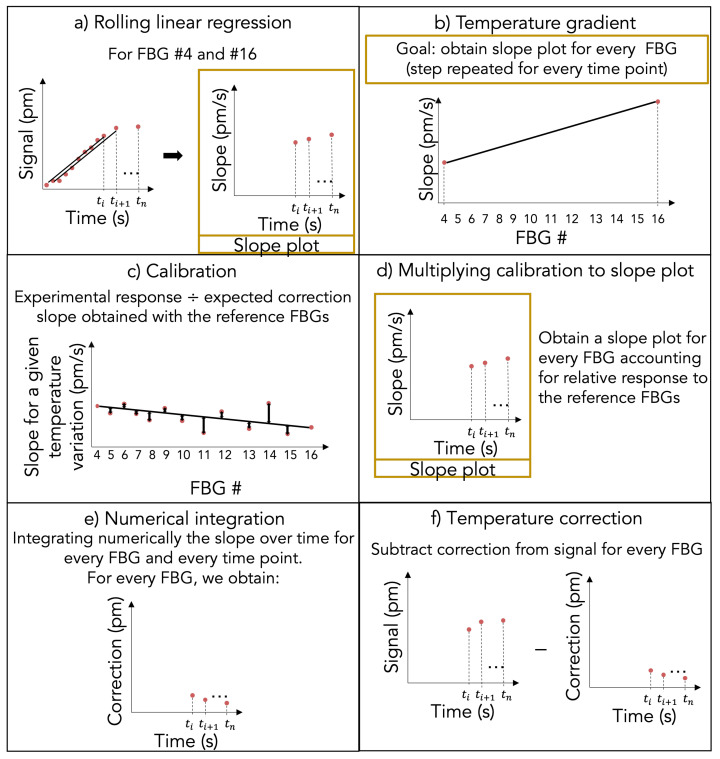
Steps to apply ITG technique: (**a**) apply a rolling linear regression to the raw data; (**b**) calculate the temperature gradient for each FBG at every time point (at the end of this step, the slope for temperature variation in time for every FBGs, called the slope plots, is obtained); (**c**) calculate the calibration factor for every FBG relative to the reference FBGs; (**d**) multiplying calibration to slope plots; (**e**) apply a numerical integration of the slope plots for every FBGs and every time point and (**f**) apply the temperature correction by subtracting the correction for the temperature contribution obtained in (**e**) to the raw data.

For the real-time interpolated temperature gradient technique, the detector and the data used are exactly the same as the PPD technique. The first step for this technique is to apply a rolling linear regression on the reference FBGs that are located outside of the irradiation field (Figure 2a). We used the signal of the 9 previous seconds, allowing a good linear fit while minimizing the signal smoothing, which implies that a 9 s pre-irradiation acquisition is required for this technique. The rolling linear regression limits the impact of the raw data noise on the correction technique.

For the second step, we assumed that the temperature varies linearly between the two references FBGs, which are placed on either side of the irradiation field since the detector is perpendicular to the ambient heat source (linear accelerator) (Figure 2b). Hence, for every time stamp, an expected correction slope is calculated linearly according to every FBG position compared to the reference FBGs. For example, with the 20 FBGs array prototype, if the first reference FBG (FBG #0) has a slope of 0 and the second reference FBG (FBG #19) has a slope of 19, the FBG #1 would have a slope of 1 and the FBG #18 would have a slope of 18.

The third step is to find the calibration factor for every FBG relative to the reference FBGs (Figure 2c). Since we glued the coating to the fiber with UV glue, the bonding strength may vary slightly for every FBG, which needs to be accounted for. This step is executed by acquiring the signal during ambient temperature variation. To do so, we acquired the signal for 300 s while the ambient temperature was linearly changing. We calculated the response slope over the 300 s acquisition due to temperature variation. We did a ratio of this experimental response and the expected correction slope obtained with the reference FBGs, which gives us the calibration factor relative to the reference FBGs.

At this point, a slope for every time point and every FBG is already obtained. That slope is different for every FBG at every time point and accounts for every FBG relative response to the reference FBGs (Figure 2d). From these slopes, the correction factor is obtained by integrating numerically the slope over time for every FBG and every time point (Figure 2e). Finally, the correction factor obtained for every timestamp needs to be subtracted from the raw data for every FBG in order to obtain the corrected data (Figure 2f). For our prototype, we used FBG #4 and #16 as reference FBGs as they had the highest responses to temperature of the ten FBGs located outside the irradiation field. Finally, to obtain the response in terms of dose, we calculated the theoretical response (0.070 pm/Gy) of our dosimeter with Equation (1) and constants presented in [21]. As with the previous temperature correction technique, once corrected, the 60 s pre-irradiation measurements are average and set to zero to allow fair comparison between each FBG.

## 3. Results

### 3.1. Dual Grating Technique

**Figure 3:**
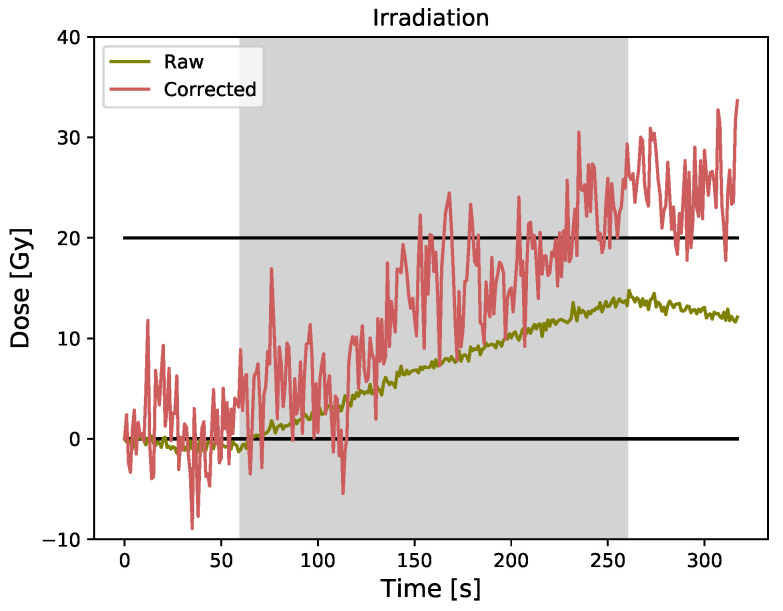
Raw and corrected data for PMMA-coated FBG obtained using the bare fiber as ambient temperature detector (dual grating technique).

The raw and corrected data obtained for a 20 Gy irradiation using a PMMA-coated FBG and a bare fiber as an ambient temperature detector is presented in Figure 3. We note a significant increase in noise on the corrected signal, which makes it hard to even see the shape of the signal. Mean values of 1±4 Gy and 25±4 Gy are obtained for the pre-irradiation and post-irradiation corrected signal. For the corrected signal, the Pearson product-moment correlation coefficient is −0.08 (*p* = 0.54) for the pre-irradiation and −0.05 (*p* = 0.69) for the post-irradiation.

**Figure 4:**
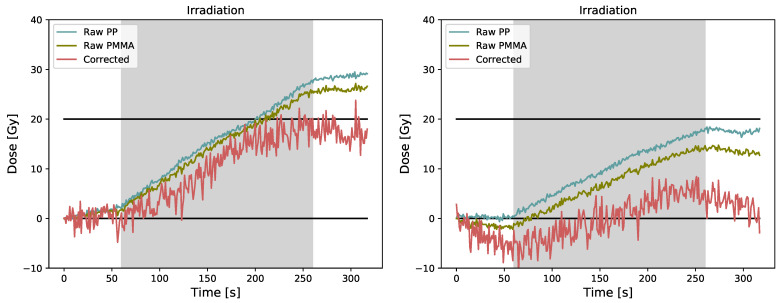
Raw and corrected data were obtained using the two polymer-embedded FBGs (dual grating technique) for (**a**) a case where both FBGs experienced the same temperature variation and (**b**) a case where both FBGs did not experience the same temperature variation.

The corrected data obtained for a 20 Gy irradiation using the two polymer-embedded FBGs are presented in Figure 4a for a case where both FBGs experienced the same temperature variation and in Figure 4b for a case where both FBGs did not experience the same temperature variation (temperature decline during pre-irradiation for PMMA, but stable for PP). Both results were obtained with the same set-up an hour apart from each other. Again, the corrected data show increased noise in both cases. Mean values of 0±2 Gy and 17±2 Gy are obtained for the pre-irradiation and post-irradiation corrected signal in Figure 4a. The Pearson product-moment correlation coefficient is 0.05 (*p* = 0.73) for the pre-irradiation and −0.20 (*p* = 0.07) for the post-irradiation. Mean values of −4±3 Gy and 3±2 Gy are obtained for the pre-irradiation and post-irradiation corrected signal in Figure 4b. The Pearson product-moment correlation coefficient is −0.71 (*p* < 0.0001) for the pre-irradiation and −0.50 (*p* < 0.0001) for the post-irradiation.

### 3.2. Pre-Irradiation and Post-Irradiation Temperature Drift (PPD)

**Figure 5:**
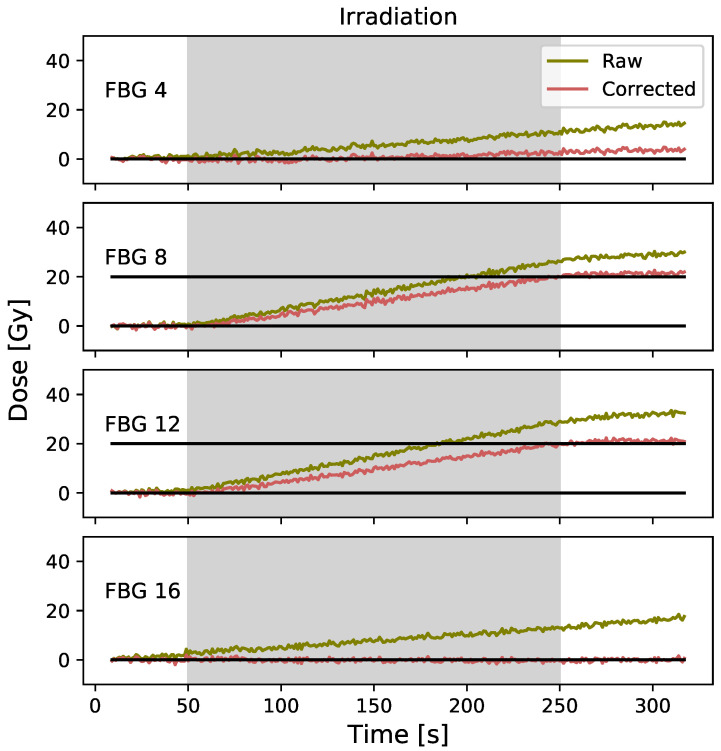
Raw and corrected data were obtained using the PPD technique. FBG #4 and #16 are located outside the irradiation field and the FBG #8 and #12 are located inside the field. It should be noted that here that only 50 s of pre-irradiation are plotted to allow easier comparison with the next technique, but the correction was generated over the 60 s of pre-irradiation acquisition.

**Figure A1:**
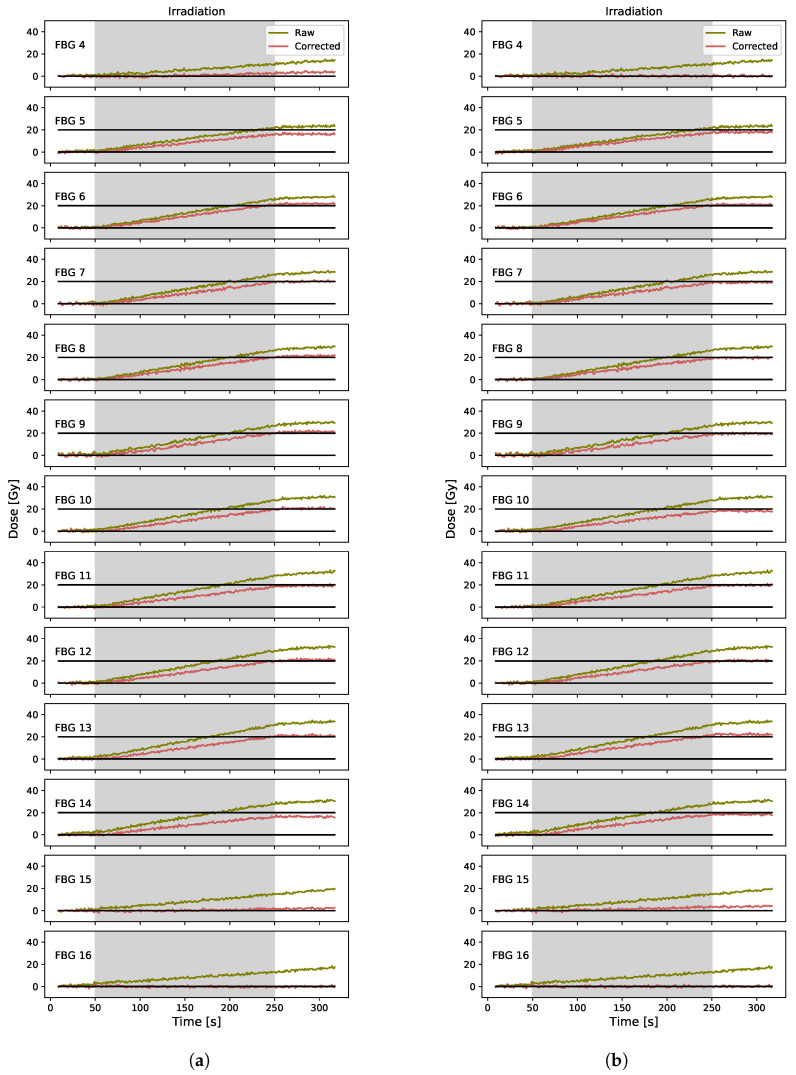
Raw and corrected data obtained using the (**a**) PPD technique and the (**b**) ITG technique.

The corrected data obtained for a 20 Gy irradiation using the PPD technique is presented in Figure 5. For the FBG #8 corrected signal, the Pearson product-moment correlation coefficient is −0.12 (*p* = 0.42) for the pre-irradiation and −0.25 (*p* = 0.05) for the post-irradiation. A mean dose value of 21.3±0.6 Gy is obtained for the post-irradiation corrected signal. For the FBG #12 corrected signal, the Pearson product-moment correlation coefficient is −0.18 (*p* = 0.21) for the pre-irradiation and 0.28 (*p* = 0.03) for the post-irradiation. A mean dose value of 21.0±0.7 Gy is obtained for the post-irradiation corrected signal. The results for FBG #4 through FBG #16 are presented in Figure A1a.

### 3.3. Real-Time Interpolated Temperature Gradient for Multi-Points Dosimetry (ITG)

**Figure 6:**
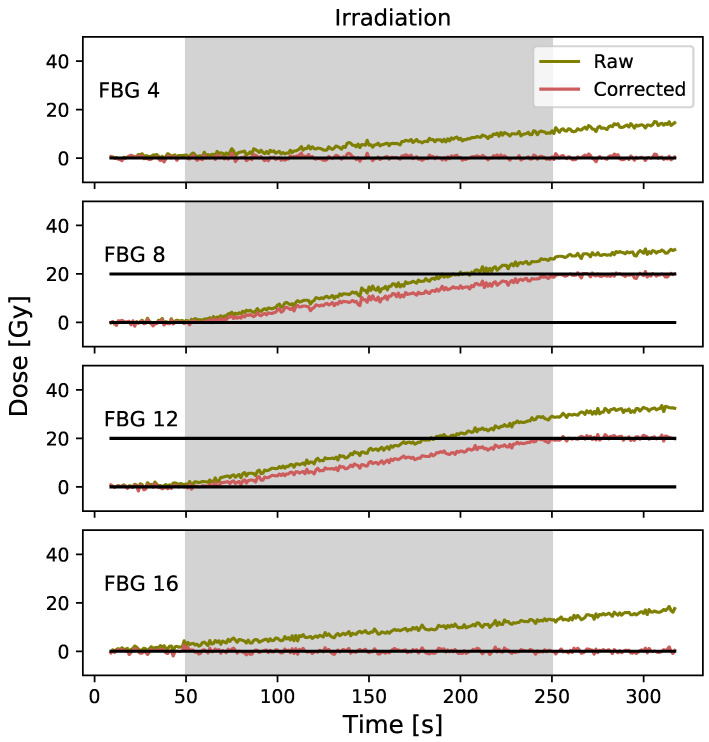
Raw and corrected data were obtained using the ITG technique. FBG #4 and #16 are located outside the irradiation field and FBG #8 and #12 are located inside the field.

The corrected data obtained for a 20 Gy irradiation using the ITG technique are presented in Figure 6. For the FBG #8 corrected signal, the Pearson product-moment correlation coefficient is 0.02 (*p* = 0.88) for the pre-irradiation and −0.01 (*p* = 0.92) for the post-irradiation. A mean dose value of 19.6±0.6 Gy is obtained for the post-irradiation corrected signal. For the FBG #12 corrected signal, the Pearson product-moment correlation coefficient is −0.02 (*p* = 0.91) for the pre-irradiation and 0.11 (*p* = 0.40) for the post-irradiation. A mean dose value of 20.3±0.6 Gy is obtained for the post-irradiation corrected signal. The results for FBG #4 to FBG #16 are presented in Figure A1b in the Appendix A.

**Figure 7:**
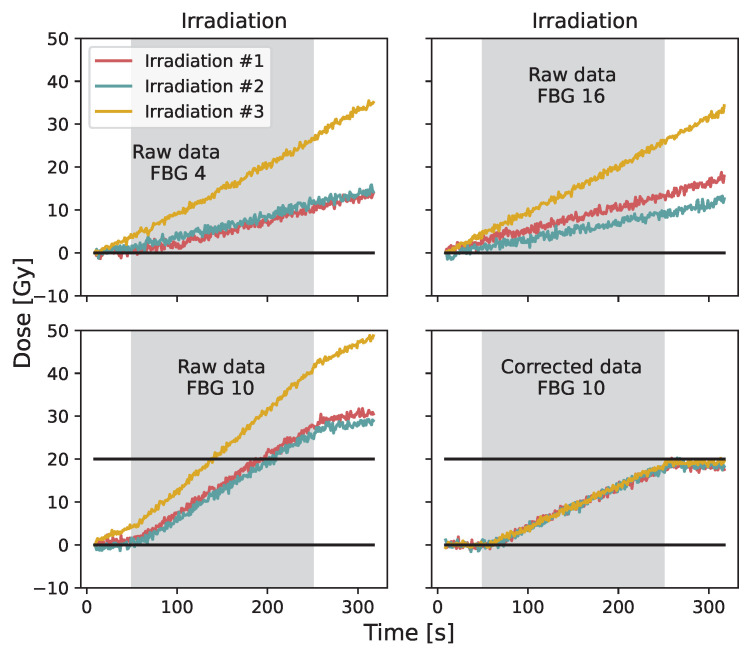
Raw data for FBG #4, #16, #10 and corrected data for FBG #10 for three different irradiation. Irradiations #1 and #2 were performed on the same day, and irradiation #3 was performed approximately one month later.

Figure 7 presents the raw data for FBG #4, #16, #10 and the corrected data for FBG #10 for three different irradiations. Irradiations #1 and #2 were performed on the same day, and irradiation #3 was performed approximately one month later. A mean dose value of 18.3±0.6 Gy, 18.6±0.6 Gy and 19.2±0.4 Gy are obtained for the post-irradiation corrected signal of irradiation #1, #2 and #3.

### 3.4. Comparison of Multi-Point Temperature Correction Techniques

**Figure 8:**
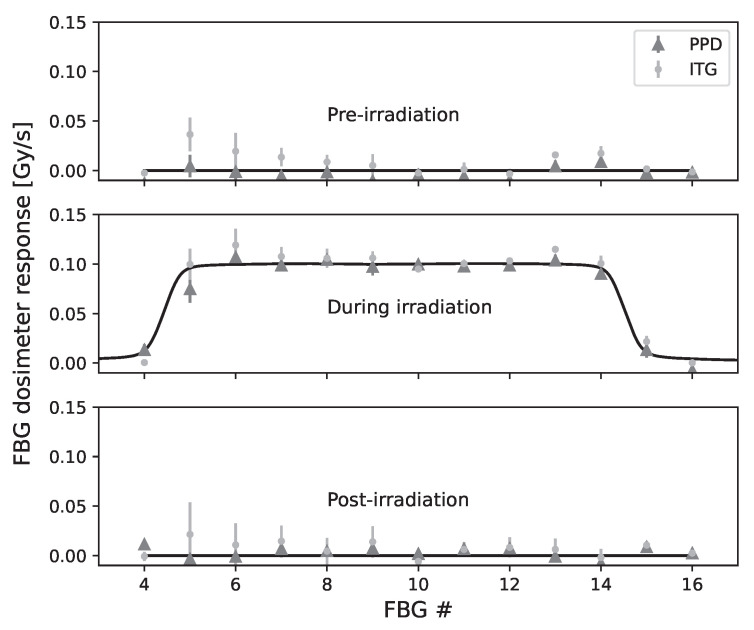
Mean slope and standard deviation over three different irradiations for every FBG in the multi-points dosimeter. The 51 s pre-irradiation, 200 s during irradiation, and the 60 s post-irradiation are presented for both the PPD technique and the ITG technique. The continuous line represents the expected values, which were measured with an ion chamber.

The mean slope and standard deviation over three different irradiations in terms of FBG numbers are presented in Figure 8. The pre-irradiation (51 s), during irradiation (200 s), and the post-irradiation (60 s) readings are presented for the PPD and ITG correction methods. During irradiation measurements, a mean difference of 0.01±0.01 Gy/s and 0.01±0.01 Gy/s are obtained obtained over 13 FBGs between corrected measurements and expected values for the PPD and the ITG, which represent an error of around 10% compared to errors reaching 200% without correction.

## 4. Discussion

### 4.1. Dual Grating Technique

Using the bare fiber as an ambient temperature detector is not suited for low-dose medical radiation because it introduces significantly more noise to the signal. To improve this technique, it would be possible to filter the corrected data, but that would disable real-time correction. However, this approach could be used for higher-dose radiation and for applications that do not require real-time measurements. Having absolute correlation coefficients that are less than 0.35 indicates that there is little if any linear correlation between the corrected signal and time [30], which is what we expect for the corrected signal.

Using the two polymer-embedded FBGs might have been suited for low-dose medical radiation, but as shown, the two detectors do not always experience the same temperature variation which is really problematic. When the temperature variation is coherent for both FBGs, the absolute correlation coefficients are less than 0.35, which indicates a low correlation between the corrected signal and time as expected. However, when both FBGs do not experience the same temperature variation, absolute correlation coefficients are 0.5 and 0.7, which indicates a moderate and a high correlation between the corrected signal and time [30], which implies that the correction failed. We think that the different temperature variations experienced by both FBGs arises from the fact that the experimental set-up is not airtight; hence, convection can occur inside the set-up and between both polymer-embedded FBGs, which breaks the temperature equilibrium. To disable convection from happening, the set-up would need to be airtight, which could be achieved with a dedicated dosimetric phantom for example. This approach is more suited for fixed gantry dose measurements.

### 4.2. Pre-Irradiation and Post-Irradiation Temperature Drift (PPD)

The expected value of 20 Gy is not included in both FBG #8 and FBG #12 post-irradiation mean dose measurements uncertainty bracket, and the absolute correlation coefficients are less than 0.35 in every case, which indicates that there is little if any linear correlation between the corrected signal and time as expected [30]. In both cases, we obtain smaller negative correlation coefficients for the pre-irradiation corrected signal and bigger positive correlation coefficients for the post-irradiation corrected signal, which indicates that the factor used to correct the signal was too big for the pre-irradiation signal but too little for the post-irradiation signal. This type of result is inherent to this correction technique since we use the mean temperature response before and after irradiation. Hence, it is not always accurate and can lead to dose–response variation when there is a discrepancy between pre-irradiation and post-irradiation temperature variation. Other than this limitation, there are two more restrictions for clinical use: the necessity to have a linear ambient temperature variation and the impossibility to do real-time dosimetry. Hence, this correction technique would be suitable mainly for quality control in radiotherapy.

### 4.3. Real-Time Interpolated Temperature Gradient for Multi-Points Dosimetry (ITG)

The expected value of 20 Gy is included in both FBG #8 and FBG #12 post-irradiation mean dose measurements uncertainty bracket, and the absolute correlation coefficients are less than 0.35 in every case, which indicates that there is little if any linear correlation between the corrected signal and time [30], which implies that the correction was successful. No pattern for the correlation coefficients is obtained, which is ideal. However, for three different irradiations, the FBG #10 does not include the expected value of 20 Gy in the post-irradiation mean dose measurements uncertainty bracket. For this FBG, we have a systematic error that translates as an over-correction which leads to a pre-irradiation and post-irradiation response smaller than zero as shown in Figure 8. This systematic error, which also occurs for FBGs 5-10 and 13, can be attributed to the calibration factor. In this work, we used only one acquisition to obtain this factor, but ideally, we would do more than one acquisition and average the results. The main limitation of this technique would lie in the assumption that temperature varies linearly between the two reference FBGs, which might not always be the case. However, we have not found any set-up configuration for radiotherapy dose measurements that would prove this assumption wrong yet.

### 4.4. Comparison of the Multi-Points Temperature Correction Techniques

The PPD technique correlation coefficients are six times larger than the ITG technique on average, which indicates a smaller linear correlation between the corrected signal and time for ITG, hence a better temperature correction. In terms of dose measurement, both techniques obtained the same difference between experimental and expected values. As mentioned previously, a systematic error was found with the ITG technique. Improving the method to measure the calibration factor could help to reduce this systematic error. The most important advantage of ITG technique is the real-time correction feature, which is impossible for the PPD technique. The PPD technique also requires a linear ambient temperature variation, which can limit its usage.

## 5. Conclusions

In this article, we set out to test different techniques to compensate for ambient temperature variations occurring during the radiotherapy treatments. We tested two types of dual grating techniques using different coatings, the PPD technique, which is commonly used for calorimetry, and we developed the ITG technique. We determined that the dual grating temperature correction technique is not ideal for radiotherapy dosimetry since it adds noise to the signal and is not identical around the linear accelerator rotation axis, but it could be used at a fixed irradiation angle for beam monitoring, for example. The PPD and ITG techniques are preferable for radiotherapy dosimetry because no noise is added to the corrected signal. For three 20 Gy irradiation, average differences of 0.01±0.01 Gy/s and 0.01±0.01 Gy/s are obtained over 13 FBGs between corrected measurements and expected values for the PPD and the ITG, which represent an error of around 10% compared to errors reaching 200% without correction. Unlike PPD, ITG allows for real-time correction, which is a net advantage for radiotherapy dosimetry. Temperature correction for medical low-dose range dosimetry is a first in our field, and it opens the door to clinical FBG dosimetry applications with a multi-points dosimeter having a submillimeter size and a customizable spatial resolution.

## Data Availability

The data presented in this study are available on request from the corresponding author.
